# Modulation of the sympathetic nervous system in youngsters by vitamin‐D supplementation

**DOI:** 10.14814/phy2.13635

**Published:** 2018-04-02

**Authors:** Rune Tønnesen, Peter Schwarz, Peter Hovind, Lars Thorbjørn Jensen

**Affiliations:** ^1^ Department of Clinical Physiology and Nuclear Medicine Rigshospitalet Glostrup Copenhagen Denmark; ^2^ Department of Endocrinology PE Research Centre of Aging and Osteoporosis Rigshospitalet Copenhagen Denmark; ^3^ Faculty of Health Sciences University of Copenhagen Copenhagen Denmark; ^4^ Department of Clinical Physiology and Nuclear Medicine University Hospital of Herlev Copenhagen Denmark

**Keywords:** Head‐up tilt table, heart rate, norepinephrine, sympathetic nervous system, vitamin D

## Abstract

The level of circulating vitamin D is known to be associated with the ejection fraction in heart failure patients and studies in rats have shown that vitamin D depletion leads to increased levels of circulating norepinephrine and decreased atrial contractility. We elucidated the effects of vitamin D supplementation on the autonomous nervous system in healthy youngsters. Thirty healthy subjects aged 18–25 years were recruited based on their serum 25‐hydroxyvitamin D (25[OH]D): 15 vitamin D insufficient (25[OH]D < 50 nmol/L) and 15 vitamin D sufficient (25[OH]D > 80 nmol/L) subjects. Both groups had vitamin D supplementation (30 microg/day) and were tested at baseline and after 30, 90, and 180 days. At each visit the serum 25‐hydroxyvitamin D was measured and the head‐up tilt test performed. Serum 25[OH]D remained stable in the vitamin D sufficient group, while the insufficient group had a significant increase (32.0–64.5 nmol/L), *P* < 0.0001. Despite the increase, the insufficient group did not reach the level of the vitamin D sufficient group within the 6 months observational period (96.1 vs 64.5 nmol/L), *P* < 0.01. Serum norepinephrine at baseline was higher in the insufficient group (mean = 1.61 nmol/L) than in the vitamin D sufficient group (mean = 0.94 nmol/L), *P* < 0.01, whereas the response to tilt was lower in the insufficient group (mean = 0.69 nmol/L) compared to the sufficient group (mean = 1.17 nmol/L), *P* < 0.01. The heart rate at rest was higher in the insufficient group (mean = 67.7 bpm) than in the vitamin D sufficient group (mean = 56.6 bpm), *P* < 0.01, for the three first visits. At the last visit no difference was found. The systolic and diastolic blood pressure differed between the groups after a month, with higher pressures in the insufficient group than in the sufficient group. Vitamin D supplementation modulates the sympathetic nervous system in healthy youngsters with low serum vitamin D. The observation might lead to a greater focus on possible prevention of cardiac disease later on in life by vitamin D supplementation early in life.

## Introduction

Heart failure is associated with low 25‐hydroxyvitamin D (25‐[OH]D) levels (Lee et al. 2008) and altered norepinephrine (NorEp) levels (Thomas and Marks 1978; Cohn et al. 1984). Vitamin D supplementation has recently been shown to improve ejection fraction in heart failure patients (Dalbeni et al. 2014) and has been associated with improvement of cardiac tone (Mann et al. 2014).

Previous studies in animals have provided valuable insight regarding the possible mechanisms behind the association. First, in Sprague‐Dawley rats (SDRs), a vitamin D‐depleted diet leads to increased circulating NorEp (Baksi and Hughes 1984), and decreased atrial contractility when stimulated with NorEp, suggesting a decreased atrial sensibility to NorEp caused by the vitamin D depletion (Baksi and Hughes 1986). Second, a vitamin D‐free diet induced an increase in the systolic blood pressure and the mean arterial pressure in SDRs (Baksi 1988), which could be explained by the increase circulating levels of NorEp (Baksi and Hughes 1984). Shermann rats (S) fed a vitamin D‐free diet for 30 days resulted in adrenal glands with high concentration of NorEp, low phenylethanolamine‐N‐methyltransferase activity, and normal epinephrine levels.

In vivo administration of 1*α*,25‐dihydroxycholecalciferol (1*α*,25[OH]_2_D_3_) has also been found to have a cardiovascular effect. Doses of 1*α*,25[OH]_2_D_3_ in vivo lead to increased contractility of resistance arteries (measured in mesenteric artery) when stimulated with NorEp, which was found in single, short, and long‐term administration studies (Bukoski et al. 1990, 1993; Bukoski and Xue 1993; Hatton et al. 1994) in SHRs and in single and short‐term studies in Wister‐Kyoto rats (WKR) (Bukoski et al. 1990; Bukoski and Xue 1993). This positive inotropic effect is also found in Wistar rats (WR) and is most likely the result of increased Ca^2+^‐channel‐mediated Ca^2+−^influx (Bian et al. 1996). Administration of 1,25[OH]_2_D_3_ in vivo favors bolus administration over continuous administration with regard to the positive inotropic effect of 1,25[OH]_2_D_3_ (Hatton et al. 1994). In rats, the modulation of vitamin D content in the diet affects circulating NorEp concentrations, the systolic blood pressure, and the contractility of the atria. Administration of 1,25[OH]_2_D_3_ in vivo modulates the contractility of the atria and increases the contractility of the resistance arteries but does not affect the blood pressure. The link between 1,25[OH]_2_D_3_ and cardiovascular function is reproduced in healthy dogs (Jahn et al. 1991; Jespersen et al. 1998). Based on this overlap in clinical findings and results from animal studies, we hypothesized a relationship between vitamin D status, circulating NorEp and cardiovascular function. The aim of this study was to investigate the effect of increasing 25[OH]D by vitamin D + calcium supplementation on the sympathetic nervous system. We conducted head‐up tilt table (HUT) testing repeatedly during vitamin D supplementation and explored the associations between 25[OH]D, circulating NorEp, heart rate, and blood pressure before and after HUT in normal healthy young adults just differing in the level of circulating vitamin‐D.

## Subjects and Methods

The study was carried out from October 2012 to August 2014 as a single center, parallel cohort, clinical controlled trial conducted at the Department of Clinical Physiology, Nuclear Medicine & PET, Rigshospitalet Glostrup.

The Inclusion criteria were 18–25 years of age and either sex. The exclusion criteria included: pregnancy, a disease known to interfere with vitamin D, hypertension, heart or kidney disease, diabetes, epilepsy, bone disease, use of systemic anabolic steroids, or supplementation or drug treatment known to affect parameters of measurement. Each subject's respective vitamin D level is a result of their lifestyle and genes as all participants were naïve to vitamin D supplementation in any form or combination.

We recruited from a larger cohort (*n* = 99) of healthy young adults aged 18–25 years who participated in a study of S‐25[OH]D and bone mineral density (Tonnesen et al. 2016). On the basis of their S‐25[OH]D values, we recruited individuals for two groups, each with 15 participants with either vitamin D insufficiency (S‐25[OH]D < 50 nmol/L) or vitamin D sufficieny (S‐25[OH]D > 80 nmol/L.

**Figure 1:**
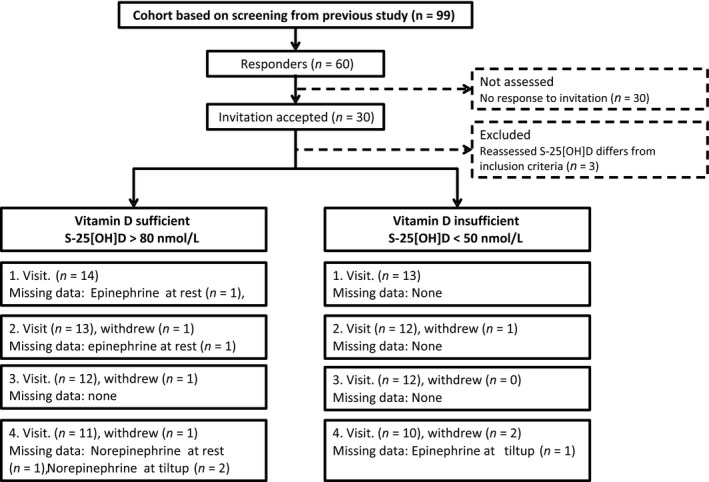
Flow diagram of recruiting and progress through the phases of a parallel‐group, investigative cohort study.

We invited 60 subjects, of whom 27 had sufficient vitamin D and 33 had insufficient vitamin D levels, by e‐mail. We halted recruitment when 15 vitamin D sufficient and 15 vitamin D insufficient subjects had accepted the invitation. The 25[OH]D level in serum was remeasured on the first day of the study to assure that 25[OH]D had not changed significantly since initial screening. Figure 1.

Written informed consent was obtained prior to participation. The local ethical committee (Ethical Committee for the Capital Region, Denmark (Region Hovedstaden)) approved this study, with ref. No. H‐1‐2012‐023.

### Study program

The program comprised four visits for evaluation of the sympathetic nervous system with a head‐up tilt test before supplementation with vitamin D, and 30 days, 90 days, and 180 days after initiation of vitamin D supplementation. All visits took place between 08.00 and 17.00; participants had fasted for 2 h prior to all visits. Height and weight were measured at start.

### Intervention

Both groups were given vitamin D_3_ + calcium supplementation. The vitamin‐D‐insufficient group was supplemented to increase the concentration of S‐25[OH]D. The vitamin D sufficient group, that acted as the control group, aimed at a steady S‐25[OH]D concentration above the calculated lower boundary.

All participants were supplied with 180 tablets of vitamin D_3_ 10 *μ*g/tablet and calcium 400 mg calcium in a personal container during the study period and were instructed to take three tablets a day for a daily dose of 30 *μ*g vitamin D_3_ + 1200 mg calcium. The participants were instructed to bring all supplied containers and remaining tablets to their next visit for compliance estimation.

### Protocol

Evaluation of the sympathetic nervous system was conducted in a quiet room with dimmed lights and a comfortable temperature. A tilt table with a footboard support was used. We used two body positions: supine and 60 degree head up tilt test. Participants were first given peripheral i.v. access for blood sampling in the supine position and attached to the tilt table with straps. Participants rested 30 min for baseline values of blood pressure, heart rate and blood samples for the assessment of S‐25[OH]D and the two catecholamines; norepinephrine (NorEp) and epinephrine (Ep), hereafter the term catecholamines refers to NorEp and Ep combined. After assessment of the baseline values, participants were tilted to 60‐degrees head up for the next 30 min. Catecholamines were sampled again after 15 min unless the subject fainted before that; if fainting occurred, the catecholamine levels were sampled at the time of the faint. After 30 min with the head up, the subject was placed in the supine position for recovery.

### Measurements

#### Sympathetic nervous system activity

The activity of the sympathetic nervous system (SNS) was assessed from the spillover of Ep and NorEp into the circulation. Blood samples were obtained in prechilled EDTA‐tubes and centrifuged at 1500 *g* at 5°C for 10 min; the samples were subsequently stored at −80 until analysis. serum NorEp and Ep were analyzed with a radio immuno assay (RIA) (2‐Cat RIA, Labor Diagnostika Nord, Nordhon Germany). The Ep range was 0.0082–8.2 nmol/L with a sensitivity of 0.0027 nmol/L and inter‐assay coefficient of variance (CV) 3.5% and intra‐assay CV 4.0%. For NorEp, the range was 0.36–35.5 nmol/L, sensitivity was 0.15 nmol/L with inter‐assay CV 2.5% and intra‐assay CV 3.2%.

#### Serum‐25‐Hydroxyvitamin D

The measurement of 25[OH]D in the serum was based on blood samples obtained in tubes with serum clot activator that were subsequently centrifuged at 1500 *g* at 10 min. The serum was frozen at −20°C until analysis with the chemiluminescent immunoassay (Liaison^®^ 25‐OH Vitamin D Total Assay; Diasorin Inc., Saluggia (Vercelli), Italy). 25‐hydroxyvitamin D was analyzed in the routine laboratory, inter‐assay CV 9.1–9.8% and intra‐assay 2.5%.

#### Hemodynamic monitoring

Noninvasively cardiovascular assessment with continous heart‐rate and beat‐to‐beat blood pressure (TaskForce:CNSystems, Graz, Austria). The coefficient of variation for the sBP and dBP was 6.8% for both. The cardiac output measured by impedance cardiography was not measured, even though the TaskForce‐system allowed it. Electrocardiogram electrodes were placed in six limb leads configuration to measure heart rate (HR). Systolic and diastolic blood pressures were measured both with beat‐to‐beat applying finger‐plethysmography and with an arm cuff.

### Statistics

Sample size for the primary outcome of catecholamines was based on the least relevant effect size of 0.5 nmol/L with an SD = 0.4 nmol/L, *α *= 0.05, and *β *= 0.80; we calculated a sample size of 12 subjects for each group. For continuous data, visual inspection was applied to determinate distribution. Unadjusted continuous data are reported as the mean ± SD when data follow a normal distribution and as median and interquartile range (IQR) when they did not. Categorical variables are summarized. Baseline values were tested using the unpaired Student's *t*‐test or *χ*2‐test.

Boundaries to detect if subjects had crossed‐over from one group to another in the time from screening to entry was calculated as limit ± 2 · CV · limit, a CV of 9.8% was used. The upper limit for vitamin‐D‐insufficient group was 50 nmol/L and the boundary calculated to 60 nmol/L. The lower limit for the vitamin‐D‐sufficient group was 80 nmol/L and boundary was calculated 64 nmol/L.

A mixed model was applied to take advantage of repeated measurements and mixed random and fixed effects and address missing data. Missing data were assumed missing at random. The fixed effects were sex and vitamin D level. Estimates are adjusted for sex and reported estimated means with 95% Cl. The level of the individuals physical activity was not significant and was excluded from the final model.

Comparison of simple effects was performed at each visit without separating data in one analysis to test for the effect of vitamin D + calcium supplementation. For all statistics (SAS^®^ Version 9.3, Cary, NC) was used. We considered a *P*‐value of 0.05 to be statistically significant.

## Results

Based on the screening values of S‐25[OH]D, we invited by e‐mail 27 and 33 subjects with sufficient and insufficient serum vitamin D, respectively. Fifteen subjects from each invited group accepted the invitation. Time from screening to study entry was not different between groups. After reassessment of S‐25[OH]D we excluded two participant with insufficiency and one with deficiency, as their S‐25[OH]D concentrations had changed significantly since screening. Not all participants completed all four visits; three in the sufficient group and three in the insufficient group dropped out. Flowchart for recruiting and progress of study is given in Figure 1.

The analysis comprised 11 men with a mean age of 20.4 ± 1.7 and 16 women with a mean age of 21.0 ± 2.2. The characteristics of the participants at baseline visit are listed in Table 1. At the baseline visit, the vitamin D insufficient group had lower S‐25[OH]D levels, lower physical activity, higher HR, higher NorEp levels compared with the sufficient group.

**Table 1:** Characteristics of the study participants at baseline by vitamin D group

Variable	Vitamin D sufficient (*N* = 14)	Vitamin D insufficient (*N* = 13)	*P*‐value
Days from screening to entry (Mean ± SD)	37.1 ± 26.9 (*N* = 14)	54.8 ± 27.1 (*N* = 13)	0.062^a^
Age (years) (Mean ± SD)	20.5 ± 2.4 (*N* = 14)	21.7 ± 2.0 (*N* = 13)	0.13^a^
S‐25[OH]D (nmol/L) (Mean ± SD)	99.9 ± 24.1 (*N* = 14)	32.0 ± 16.5 (*N* = 13)	0.0001^a^
Weight (kg) (Mean ± SD)	70.1 ± 11.1 (*N* = 14)	78.4 ± 15.4 (*N* = 13)	0.23^a^
Height (m) (Mean ± SD)	176 ± 9.7 (*N* = 14)	174.2 ± 6.0 (*N* = 13)	0.56^a^
Calcium (pmol/L) (Mean ± SD)	1.24 ± 0.04 (*N* = 13)	1.24 ± 0.02 (*N* = 12)	0.58^a^
iPTH (pg/mL) (Median (IQR))	39 (16‐83) (*N* = 13)	56 (29–130) (*N* = 12)	
Systolic BP (rest)(mmHg) (Mean ± SD)	116 ± 11.2 (*N* = 14)	123 ± 15.2 (*N* = 13)	0.11^a^
Diastolic BP (rest)(mmHg) (Mean ± SD)	66.1 ± 6.3 (*N* = 14)	72.8 ± 12.0 (*N* = 13)	0.11^a^
Heart rate (rest)/bpm (Mean ± SD)	56.6 ± 7.2 (*N* = 14)	67.7 ± 10.1 (*N* = 13)	0.007^a^
Norepinephrine, rest (nmol/L)(Mean ± SD)	0.9 ± 0.3 (*N* = 14)	1.6 ± 0.5 (*N* = 13)	0.0002^a^
Epinephrine, rest (nmol/L)(Mean ± SD)	0.1 ± 0.0 (*N* = 13)	0.1 ± 0.0 (*N* = 13)	0.66^a^
Sex			
Women	10	6	0.18^b^
Men	4	7	
Tobacco (last week)			
No	13	10	0.24^b^
Yes	1	3	
Alcohol consumption (last week)			
No	8	6	0.57^b^
Yes	6	7	
Physical activity (h/week)			
0–2	1	8	0.011^b^
2–7	5	2	
7 and above	8	3	

a Based on Mann–Whitney test.

b Based on Chi‐square test.

The period of vitamin D + calcium supplementation (median [IQR]) was 184 [180–200] days for the insufficient group and 183 [179–200] days for the control group. Compliance (mean) for the insufficient and sufficient group, respectively was from visit one to two; 83% and 81%, from visit two to three; 84% and 82%, and from visit three to four 90% and 87%.

**Figure 2:**
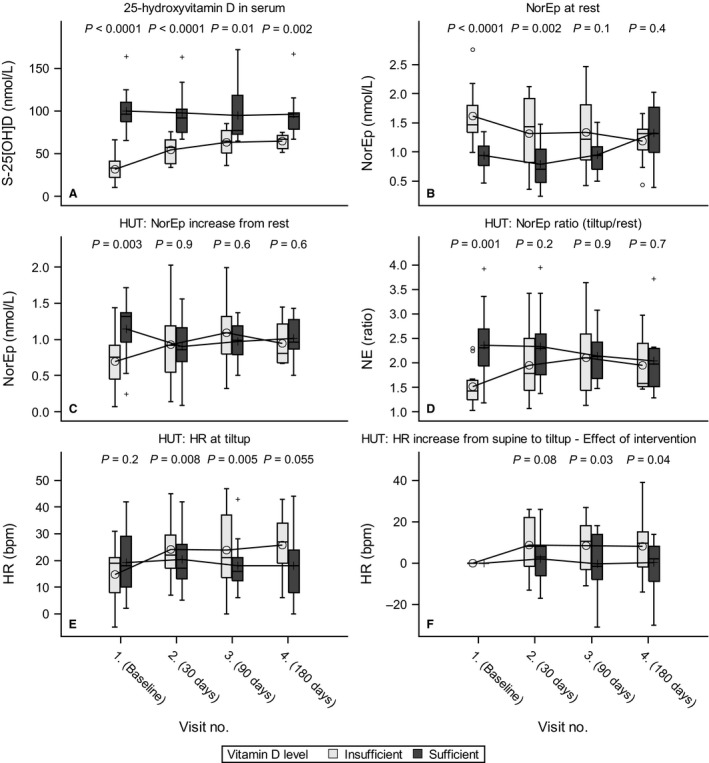
(A) 25‐hydroxyvitamin D for groups; (B) plasma norepinephrine (NorEp) at rest; (C) Head up tilt test (HUT) increase in NorEp when raised from supine(rest) to standing (tiltup); (D) HUT ratio for NorEp at tiltup over rest; (E) HUT heart rate (HR) in beats per minute at tilt up; (F) HUT increase in HR from rest to tiltup ‐ Effect of intervention is calculated as the response values subtracted from the values from the first visit, for each subject.

From the baseline visit to the fourth visit, the vitamin D insufficient group increased their S‐25[OH]D by 32.3 (21.6–42.9)nmol/L, *P* < .0001, and the vitamin D levels in the sufficient group remained steady with a change in S‐25[OH]D of −5.9 (−16.1 to 4.3) nmol/L, *P* = 0.25. The vitamin D sufficient group remained higher in S‐25[OH]D levels at all visits compared to the insufficient group, *P* = 0.0001, see Figure 2A.

### Epinephrine and Norepinephrine

NorEp at rest was significantly higher in the insufficient group at the first and second visits compared to the sufficient group, with an estimated difference of 0.65 (0.35–0.96) nmol/L, *P* < .0001 and 0.50 (0.08–0.91) nmol/L, *P* = 0.02, respectively. At the third visit, the difference was insignificant and had further diminished at the fourth visit; see Figure 2A for the unadjusted values and Figure 2B for the plot of adjusted values. Stimulation with HUT led to an increase in NorEp; at the first visit, the increase was significantly higher in the sufficient group 1.17 (0.95–1.38) nmol/L compared with the insufficient group 0.69 (0.48–0.91) nmol/L, *P* = 0.003. At visits two through four, there was no significant difference in the NorEp increase between the groups; for the HUT‐induced increase in NorEp, see Figure 2C. The effect of vitamin D + calcium supplementation on the NorEp changes induced by HUT is present from visits two through four; see Table 2 for values and Figure 2D for the trend line.

**Table 2:** Mean ± SD for s‐25[OH]D, NorEp, heart rate, systolic and diastolic blood pressure at all four visits

		1. (Baseline)	2. (30 days)	3. (90 days)	4. (180 days)
S‐25[OH]D (nmol/L)	Suff.	99.9 ± 24.1	97.8 ± 27.6	94.4 ± 33.2	96.1 ± 27.1
	Insuff.	**32.0 ± 16.5	**54.1 ± 15.0	**63.2 ± 16.6	**64.5 ± 8.6
Norepinephrine (nmol/L), rest	Suff.	0.94 ± 0.25	0.79 ± 0.39	0.95 ± 0.32	1.33 ± 0.50
	Insuff.	**1.61 ± 0.48	**1.31 ± 0.61	1.34 ± 0.61	1.19 ± 0.37
Norepinephrine delta (nmol/L), rest to tilt up	Suff.	1.14 ± 0.39	0.90 ± 0.38	0.97 ± 0.27	1.02 ± 0.28
	Insuff.	**0.69 ± 0.41	0.93 ± 0.50	1.09 ± 0.50	0.94 ± 0.30
Heart rate (bpm), rest	Suff.	56.6 ± 7.2	56.7 ± 8.0	58.3 ± 12.5	61.2 ± 8.1
	Insuff.	**67.7 ± 10.1	**68.5 ± 9.9	**68.8 ± 15.2	65.7 ± 11.8
Heart rate delta (mmHg), rest to tilt up	Suff.	19.3 ± 12.3	20.3 ± 11.0	17.9 ± 10.0	17.9 ± 12.0
	Insuff.	14.8 ± 10.7	24.2 ± 11.4	23.8 ± 14.2	25.7 ± 11.7
Systolic BP (mmHg), rest	Suff.	116 ± 11.2	114 ± 11.8	109 ± 11.5	114 ± 6.7
	Insuff.	123 ± 15.2	**130 ± 16.1	120 ± 22.0	122 ± 11.8
Systolic BP delta (mmHg), rest to tilt up	Suff.	11.7 ± 8.0	7.8 ± 14.3	11.3 ± 9.6	12.0 ± 5.6
	Insuff.	12.4 ± 10.7	*16.6 ± 7.7	9.6 ± 7.4	9.7 ± 8.1
Diastolic BP (mmHg), rest	Suff.	66.1 ± 6.3	65.0 ± 9.2	66.6 ± 7.9	66.8 ± 5.3
	Insuff.	72.8 ± 12.0	**75.7 ± 12.9	68.8 ± 15.9	71.5 ± 7.7
Diastolic BP delta (mmHg), rest to tilt up	Suff.	20.3 ± 9.4	11.1 ± 13.7	14.8 ± 8.4	15.5 ± 7.6
	Insuff.	15.8 ± 10.2	**24.6 ± 6.4	17.3 ± 7.5	15.8 ± 7.6

Vitamin D sufficient group (Suff.) Vitamin D insufficient group (Insuff.)

Significant differences between groups are marked with * for (*P* < 0.05) and ** for (*P* < 0.01).

NorEp is the major driver of change in the levels of catecholamines rather than Ep, and this change accounts for both values at rest and in response to HUT stimulation. No significant changes in epinephrine between and within groups were found neither at rest nor after HUT stimulation.

### Heart rate

At rest, the mean heart rate was significantly higher from the first to the third visit in the sufficient group compared to the insufficient group, and at visit four, the between group difference had disappeared. Stimulation with HUT led to an increase in HR in both groups as expected. In the insufficient group, HUT at the first visit led to an increase in 14.7 (8.2–21.2) bpm. From visit one to two, it increased 9.2 (1.4–17.1) bpm; *P* = 0.02–23.9 (17.2–30.6) bpm. At the second visit, the difference in bpm remained the same as for the subsequent visits. For the sufficient group, the increase in HR when stimulated with HUT remained stable from the first through the last visits; see Table 2 and Figure 2E. The treatment effect on heart rate response to HUT was present at visit two and remained stable through to visit four; see Figure 2F.

### Blood pressure

Systolic blood pressure at rest in the supine position was significantly higher 15.6 (4.3–28.0) mmHg, *P* = 0.008 for the insufficient group at visit two after 30 days compared to the sufficient group. For all other visits, there was no difference between the groups. After HUT stimulation, the increase in systolic blood pressure was significantly higher for the insufficient group at visit two compared to the sufficient group, with increases of 16.5 (11.1–22.0) mmHg and 8.1 (2.8–13.4), *P* = 0.03, respectively.

Diastolic blood pressure at rest in the supine position was significantly higher [11.5 (3.2–19.7) mmHg, *P* = 0.007] at visit two in the insufficient group compared to the sufficient group, whereas the difference was insignificant for all other visits. HUT stimulation at visit two resulted in a diastolic blood pressure in the insufficient group of 99.8 (91.7–107.9) mmHg and 75.5 (67.6–83.5) mmHg for the sufficient group, a difference of 24.3 (12.9–35.7) mmHg, *P* < 0.0001. At the first, third and fourth visits, the HUT induced an increase in diastolic blood pressure that was insignificant.

## Discussion

The study show an association between S‐25[OH]D and the sympathetic response to HUT stimulation for several parameters, mainly interpreted to be caused by the vitamin D + calcium supplementation affecting the SNS by increasing the NorEp release to the circulation. This giving rise to a higher response of the heart rate in the vitamin D insufficient group compared with the sufficient group. The outcome is not adjusted for daily physical activity as it was insignificant.

Even after half a year of vitamin‐D supplementation, the insufficient group did not reach the level of circulating vitamin‐D as in the control group. The daily supplementation of 30 microg vitamin‐D is the normal recommended for adults in Denmark during wintertime and we did not intend to add more than normal accepted doses to normal youngsters. Given a higher dose of vitamin‐D might have increase the level in the insufficient group, but other factors might influence the individual′s level, that is, genetic dispositions.

NorEp was strongly associated with S‐25[OH]D at rest; similar results have been reported in diet studies of rats (Baksi and Hughes 1984). The effect of a vitamin D_3_‐depleted diet on NorEp at rest is reversible in rats (Brion et al. 1978a). The effects of increasing S‐25[OH]D by vitamin D + calcium supplementation on NorEp in rats and humans at rest are in line. Modulation of the NorEp response to HUT stimulation during reversal of vitamin D insufficiency has, to the best of our knowledge, not been reported before.

Modulation of the heart rate response to HUT stimulation through increase in S‐25[OH]D by vitamin D + calcium supplementation has, to our knowledge, not been reported in humans. Furthermore, it is of interest that our study was conducted in normal young adults. In rats, a vitamin D_3_‐depleted diet leads to decreased atrial contractility and vasomotor response when stimulated with NorEp (Baksi and Hughes 1986; De Novellis et al. 1994); this advocates for an effect of vitamin D depletion on cardiovascular tissue. We did not stimulate with NorEp but with HUT, which leads to increased serum NorEp, and our modulation of the heart rate response could result in both an effect on NorEp release and an effect on cardiovascular tissue caused by the increase in S‐25[OH]D as a result of vitamin D supplementation + calcium. We had no intention to be able to distinguish between central and peripheral effects on the autonomous nervous system of vitamin‐D supplementation. Most likely, the main effect is on the central parts of the SNS, but peripheral effect cannot be excluded for the time being.

## Conclusion

This study supports our hypothesis that vitamin D supplementation given to vitamin D insufficient young adult modulate their sympathetic in rest and after HUT stimulation. The mechanism behind the modulation of the response from the sympathetic nervous system is unknown, but in line with animal studies. No beneficial effects of vitamin D supplementation were found in the vitamin D sufficient group. Future trials of vitamin D_3_ or 1,25‐dihydroxyvitamin D supplementation on the SNS regulatory mechanisms are needed.

We conclude that supplementation of vitaminD and calcium in normal youngsters with low S‐25[OH]D can modulate the sympathetic nervous system giving rise to alterations in blood pressure and heart rate. The observation might be useful in planning the prevention of cardiac diseases as the vitamin D level in normal young human beings has not been taken into consideration. The clear effect on the autonomous nervous system of vitamin‐D supplementation calls for population studies on the impact of low or normal circulating levels of vitamin‐D on cardiovascular diseases later in life. On the other hand, the absence of side effects of vitamin‐D supplementation in general opens for interventional studies, adding vitamin‐D to food in general. This should be in a controlled environment to be ethical acceptable.

### Strength and limitations

One of the strengths of this study is the repeated measurement, as it gives insight to the time perspective in the modulation of the sympathetic response to vitamin D supplementation. The investigation of healthy young adults who are free of diseases and conditions is strength of the study, as it gives a clearer view of the effects with no confounders as hypertension or cardiac failure.

We have used tablets with vitamin D_3_ and calcium because it is a commonly used form of supplementation. It is well known that the prohormone 25‐hydroxyvitamin D does not in itself exert physiological effects, as that role belongs solely to 1,25‐dihydroxyvitamin D (1,25[OH]D). We have not measured 1,25‐dihydroxyvitamin D levels, which is a weakness of the study, as 1,25‐dihydroxyvitamin D has been shown to affect contractility by increasing sensitivity to NorEp of cardiovascular tissues (Bukoski et al. 1993) and to affect cardiac output in both dogs and humans (Jahn et al. 1991; Jespersen et al. 1998). Measurement of 1,25‐[OH]D might have given better insight into the physiology behind the effects of vitamin D_3_ supplementation. Measurement on 1,25[OH]D in serum might not reflect the intracellular concentration of 1,25[OH]D as some tissues possess the ability to synthesize 1,25[OH]D itself (Zehnder et al. 2001). Since 25[OH]D circulates bound to vitamin D‐binding protein, another solution could be to estimate free 25[OH]D and bound fraction. The free unbound 25[OH]D could depict the available substrate to a higher degree, than serum 25[OH]D as we have measured which is composed of both free and bound forms of 25[OH]D (Jones 2008). Secondly it is a weakness that the supplementation included both vitamin D_3_ and calcium. It cannot be excluded that calcium could play a role in regulation of serum NorEp, but our results does not justify such a role for calcium, as calcium was indifferent at baseline visit, but norepinephrine level was not.

The mechanisms behind the effect of vitamin D_3_ supplementation on NorEp at rest were beyond the scope of this experiment. Such mechanisms in humans might possibly involve the activity of phenylethanol‐N‐methyltransferase and affect the intra‐adrenal storage of NorEp, as a vitamin D‐depleted diet can lead to this condition in rats, and the condition is reversible with supplementation (Brion et al. 1978a,b).

To hopefully curb the seasonal variation in S‐25[OH]D expected to affect our measurements, we used a group with a stable concentration of S‐25[OH]D to act as a control group. A better way of reducing bias might have been a four arm study with the vitamin D insufficient and sufficient groups subdivided into two groups each, where subjects would be randomized to either treatment or placebo. In such a four‐arm study, the concentration of S‐25[OH]D in the placebo groups would fluctuate seasonally, and such a setup should cover a whole season of 12 months. Alternatively a two arm study with start at summer at zenith for S‐25[OH]D and follow‐up 30, 90, and 180 days, would ensure that S‐25[OH]D would be different in the two groups, as the placebo group would follow the trajectory of the seasonal variation.

These results could give insight into some of the pathophysiological effects of vitamin D insufficiency and diseases where NorEp levels are affected and treatment of vitamin D insufficiency is beneficial. However, heart failure is known for the association between NorEp and mortality (Cohn et al. 1984), and the beneficial effect of vitamin D supplementation(Dalbeni et al. 2014) could be related to the effect of vitamin D supplementation on NorEp. Our results might have limitations if transposed to populations with disease. The modulator effect on the sympathetic nervous system of vitamin D supplementation could be too small to have an impact or the pathophysiology behind the disease blocks the pathway, which is affected by the supplementation.

## Conflict of Interest

None declared.
